# Agreement Between Non-Cycloplegic Photorefraction and Retinoscopy in Pediatric Refraction

**DOI:** 10.3390/life16040678

**Published:** 2026-04-16

**Authors:** Ana Roque, Amélia Fernandes Nunes, Henrique Nascimento, NIAOO Group, Clara Martinez-Perez

**Affiliations:** 1 School of Administration, Engineering and Aeronautics (EGEA), Instituto Superior de Educação e Ciências de Lisboa (ISEC Lisboa), Alameda das Linhas de Torres, 179, 1750-142 Lisboa, Portugal; ana.roque@iseclisboa.pt (A.R.); henrique.nascimento@iseclisboa.pt (H.N.); dgid@iseclisboa.pt (NIAOO Group); 2 Center for Research in Health Sciences (CICS), University of Beira Interior, 6200-506 Covilhã, Portugal; amnunes@ubi.pt; 3 Applied Physics Department (Optometry Area), Facultade de Óptica e Optometría, Universidade de Santiago de Compostela, 15705 Santiago de Compostela, Spain

**Keywords:** pediatric optometry, photorefraction, retinoscopy, accommodation, refractive error, vision screening

## Abstract

Accurate assessment of refractive error in children is essential for clinical decision-making, yet agreement between non-cycloplegic techniques remains uncertain, particularly due to differences in accommodative demand. This study evaluated the agreement between static retinoscopy and handheld photorefraction for measuring spherical power, cylindrical power, and spherical equivalent in children aged 4–16 years and assessed whether agreement varied by refractive status. In this cross-sectional observational study, 193 children underwent objective refraction during a single visit using non-cycloplegic static retinoscopy (distance fixation) and handheld infrared photorefraction (~1 m fixation). Inter-method differences were analyzed using Bland–Altman plots, intraclass correlation coefficients (ICCs), mean absolute error (MAE), and non-parametric tests. Photorefraction showed a statistically significant myopic shift compared with retinoscopy for spherical power (−0.16 D), cylindrical power (−0.24 D), and spherical equivalent (−0.28 D). Agreement was moderate in statistical terms for spherical equivalent (ICC = 0.73) and spherical power (0.71), and lower for cylindrical power (0.46); however, wide limits of agreement indicate clinically relevant variability. MAE for spherical equivalent was 0.80 D overall, with 45.1% of measurements within ±0.50 D, and varied by refractive status, being lowest in emmetropic eyes and higher in hyperopic and myopic eyes. These findings indicate that, under non-cycloplegic conditions, photorefraction shows modest mean differences but substantial individual variability, likely influenced by differences in accommodative demand between techniques. While suitable for pediatric vision screening, photorefraction should not be considered interchangeable with retinoscopy for individual refractive assessment.

## 1. Introduction

Accurate assessment of refractive errors is a fundamental component of optometric and ophthalmological practice, particularly in pediatric populations, where uncorrected or inaccurately corrected refractive errors may negatively affect visual development and long-term ocular health. Several techniques are available to evaluate refractive status, including subjective manifest refraction, retinoscopy, and automated objective methods. While subjective manifest refraction is widely regarded as the gold standard for spectacle prescription, it is time-consuming and requires a high level of patient cooperation, which can limit its applicability in children and high-volume clinical settings [1]. Consequently, objective techniques play a central role in pediatric refractive assessment [2].

Cycloplegic retinoscopy is accepted as the reference standard for objective refractive evaluation in children, as cycloplegia effectively eliminates accommodation and allows accurate detection of latent hyperopia and astigmatism [3,4]. However, retinoscopy is a highly operator-dependent technique that requires considerable clinical expertise and may be time-consuming, which can limit its feasibility in routine clinical practice, particularly in less cooperative pediatric patients [5,6,7]. Interobserver variability represents an additional limitation, especially when performed by clinicians with differing levels of experience [7]. For this reason, the present study focuses on non-cycloplegic conditions, reflecting routine clinical and screening practice.

To address these limitations, automated refraction techniques have been increasingly adopted in both clinical and screening contexts. Autorefraction is generally faster, easier to perform, and better tolerated by pediatric patients, contributing to its widespread use [6,8,9]. In addition, handheld photorefraction devices (photoscreeners) have gained prominence due to their portability, rapid measurement capability, and suitability for large-scale screening programs [10,11,12,13]. However, the accuracy of these devices under non-cycloplegic conditions remains a matter of debate. Previous studies have reported that non-cycloplegic measurements may underestimate hyperopia and overestimate myopia due to residual accommodative responses, particularly in children with high accommodative reserve [13,14,15,16].

Nevertheless, despite their increasing use, there is still a lack of consistent evidence regarding the agreement between handheld photorefraction and non-cycloplegic retinoscopy in pediatric populations. In particular, it remains unclear whether this agreement is consistent across different refractive errors, which limits their clinical applicability.

Comparative studies assessing agreement between retinoscopy and automated refraction have yielded inconsistent findings, especially in pediatric populations [16,17,18]. These discrepancies may be explained by methodological differences, including device type, examiner experience, and variations in accommodative control. Furthermore, several studies have highlighted limitations in the estimation of cylindrical power and astigmatic components when using handheld devices, which may affect their clinical reliability [19,20].

Beyond diagnostic accuracy, the potential consequences of imprecise refractive assessment are clinically relevant. There is increasing evidence linking accommodative effort with myopia progression, suggesting that minus overcorrection may contribute to its development or worsening in children [21,22]. Consequently, prescriptions based on non-cycloplegic automated measurements may inadvertently increase accommodative demand, reinforcing the need for reliable refractive assessment in pediatric eye care [23,24].

This study aimed to assess the agreement between non-cycloplegic static retinoscopy and handheld photorefraction for measuring spherical power, cylindrical power, and spherical equivalent in children aged 4–16 years, and to evaluate whether agreement varies according to refractive status.

## 2. Materials and Methods

### 2.1. Study Design

This was a cross-sectional observational study. The research adhered to the principles of the Declaration of Helsinki and was approved by the Ethics Committee of the Higher Institute of Education and Sciences (ISEC), Lisbon, Portugal (approval number: CE/2022/10/24). Parents or legal guardians of all participants provided written informed consent prior to inclusion in the study.

### 2.2. Study Population

The study population comprised participants who attended routine optometric examinations in the Lisbon metropolitan region (Portugal). Data collection was conducted between October 2025 and January 2026. A total of 193 participants with complete and valid objective refractive measurements were included in the analysis.

Only participants for whom both retinoscopy and photorefraction measurements were successfully obtained were considered eligible. Participants presenting ocular media opacities, retinal abnormalities, or any condition that could interfere with reliable objective refractive assessment were excluded. Measurements with insufficient quality or incomplete data were also excluded. Participants with accommodative or binocular vision disorders were not specifically assessed or excluded.

Each participant was assessed only once during the study period, with no repeated measurements.

### 2.3. Procedures

Objective refractive assessment included non-cycloplegic static retinoscopy and handheld photorefraction, reflecting routine clinical practice. All examinations were performed during a single visit under standardized lighting conditions by experienced optometrists. Only a single measurement per method was recorded for each participant, which may limit the assessment of measurement repeatability and increase measurement variability.

Both techniques were performed sequentially during the same visit, and examiners were not masked to the results of the alternative method; this may have introduced measurement bias and potential order effects. This approach reflects real-world clinical conditions, where masking and repeated measurements are not routinely performed. The order of testing was fixed, following routine clinical practice. The two techniques differed in fixation distance (approximately 4 m for retinoscopy and 1 m for photorefraction), which may act as a methodological confounder by inducing different accommodative demands and potentially contributing to systematic differences between measurements, including a myopic shift in photorefraction.

#### 2.3.1. Non-Cycloplegic Static Retinoscopy

Non-cycloplegic retinoscopy was performed using a HEINE Beta 200 streak retinoscope (HEINE, Gilching, Germany) [25] at a fixed working distance of 67 cm. Participants were instructed to fixate on a high-contrast, non-accommodative distant target positioned at approximately 4 m to minimize accommodative response during the examination. The examiner observed the movement of the retinal reflex while sweeping the retinoscope streak across the pupil.

The corresponding working distance lens (1.50 D) was subtracted from the final neutralization value. The direction and speed of the reflex movement were evaluated to determine refractive status. Trial lenses were introduced in front of the eye to neutralize the reflex using the plane-mirror mode of the retinoscope: with-motion was neutralized using positive lenses, consistent with hyperopia or low myopia relative to the working distance, whereas against-motion was neutralized using negative lenses, consistent with myopia exceeding the working distance. Cylindrical lenses were used to determine the magnitude and orientation of astigmatic components. Neutralization was achieved when the retinal reflex showed no apparent movement.

Spherical and cylindrical refractive components were recorded in negative-cylinder notation following standard retinoscopic techniques. Neutralization was determined by a single examiner, and no independent verification or second examiner assessment was performed.

#### 2.3.2. Photorefraction

Photorefraction was obtained using the 2WIN (Adaptica, Italy, Padova) handheld device [26], which employs binocular infrared eccentric photorefraction to estimate refractive error under non-cycloplegic conditions. In this study, the term “photorefraction” refers specifically to handheld infrared eccentric photorefraction performed using a photoscreener device. The device was positioned at the manufacturer-recommended working distance of approximately 1.0 m, facing the participant.

Participants were instructed to look at the fixation target provided by the device, which facilitates binocular fixation and pupil alignment. The device emits infrared light reflected by the retina and captured by an internal camera system. The distribution of reflected light across the pupil is analyzed by the device’s software to estimate spherical and cylindrical refractive components.

Measurements were performed under dim ambient lighting conditions to optimize pupil size and fixation stability. Only measurements deemed valid by the device software were included, defined as successful binocular capture with appropriate pupil detection and absence of warning indicators related to alignment, fixation, or measurement reliability. Therefore, individual measurement attempts and excluded measurements were not separately recorded. Device reliability scores were not systematically documented, which precludes assessment of potential exclusion bias and measurement reliability.

#### 2.3.3. Data Recording and Refractive Classification

For each participant, spherical power, cylindrical power, and spherical equivalent (SE) were recorded for both retinoscopy and photorefraction in negative-cylinder notation. The spherical equivalent was calculated as SE = sphere + (cylinder/2).

Refractive status classification was based on retinoscopic spherical equivalent values and followed commonly accepted clinical thresholds consistent with the definitions proposed by the International Myopia Institute [4]: myopia (SE ≤ −0.50 D), hyperopia (SE ≥ +0.50 D), and emmetropia (SE between −0.50 D and +0.50 D). Refractive classification was based on non-cycloplegic retinoscopy and may therefore underestimate latent hyperopia.

### 2.4. Statistical Analysis

Statistical analysis was performed using R software (version 4.4.2). All analyses were conducted using data from the right eye (OD) only, in order to avoid inter-eye correlation. Normality of refractive variables and inter-method differences was assessed using the Shapiro–Wilk test.

Continuous variables are summarized using mean ± standard deviation (SD), median and interquartile range (IQR), and minimum–maximum range, as appropriate. Inter-method differences were calculated as the difference between photorefraction and retinoscopy for spherical power, cylindrical power, and spherical equivalent.

Differences between retinoscopy and photorefraction were evaluated using the Wilcoxon signed-rank test, with statistical significance set at *p* < 0.05. Agreement between methods was assessed using Bland–Altman analysis, reporting the mean inter-method difference and the 95% limits of agreement (LoAs), calculated as mean difference ± 1.96 × SD of the differences.

To quantify absolute inter-method discrepancies, the mean absolute error (MAE) was calculated for each refractive parameter.

Potential proportional bias was evaluated by linear regression analysis, with inter-method differences plotted against the mean of both methods for each refractive parameter. Method agreement was further assessed using intraclass correlation coefficients (ICCs) based on a two-way random-effects model with absolute agreement. Interpretation of ICC values followed commonly accepted criteria [27]: values < 0.50 were considered poor, 0.50–0.75 moderate, 0.75–0.90 good, and >0.90 excellent.

Subgroup analyses were performed according to refractive status, and within each group, Bland–Altman metrics, MAE, and concordance rates within predefined clinically acceptable thresholds (±0.50 D and ±1.00 D) were calculated.

## 3. Results

A total of 193 participants (108 females and 85 males), aged between 4 and 16 years, were included in the analysis. Table 1 presents descriptive statistics for refractive parameters measured by retinoscopy and photorefraction.

Myopia was more prevalent in males (*n* = 26) than in females (*n* = 14), whereas emmetropia was slightly more common in females (*n* = 28) than in males (*n* = 21); however, no statistically significant association was found between sex and refractive status (χ^2^ = 4.36, df = 2, *p* = 0.113). Participant-level anonymized data are available as Supplementary Material S1.

**Figure 1:**
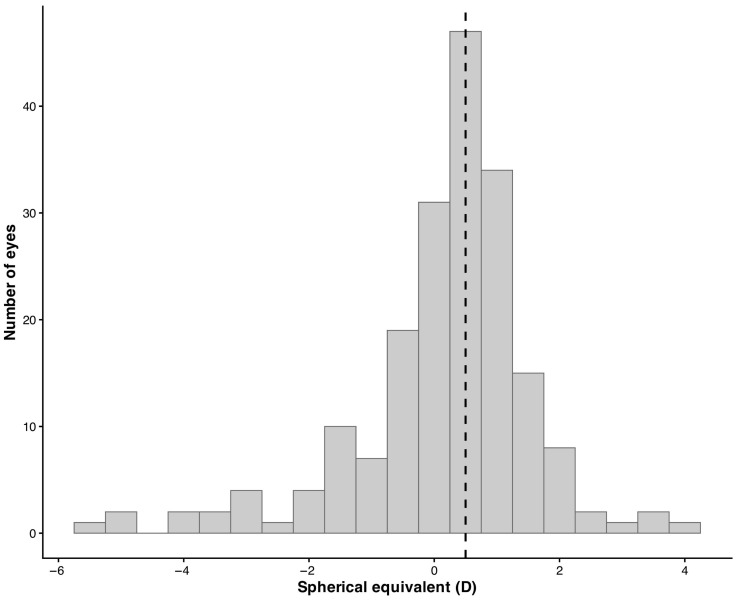
Frequency distribution of spherical equivalent values measured by retinoscopy in the study population. The dashed vertical line represents the median value.

Overall, spherical equivalent values measured by retinoscopy showed a wide distribution across the refractive spectrum, with a predominance of low refractive errors. The distribution of spherical equivalent values obtained by retinoscopy is illustrated in Figure 1, which shows a concentration of measurements around emmetropic and low ametropic values, with fewer observations at higher refractive errors.

Overall, agreement between methods was characterized by small mean differences but wide limits of agreement across all refractive parameters, which exceeded predefined clinically acceptable thresholds (±0.50 D and ±1.00 D).

**Figure 2:**
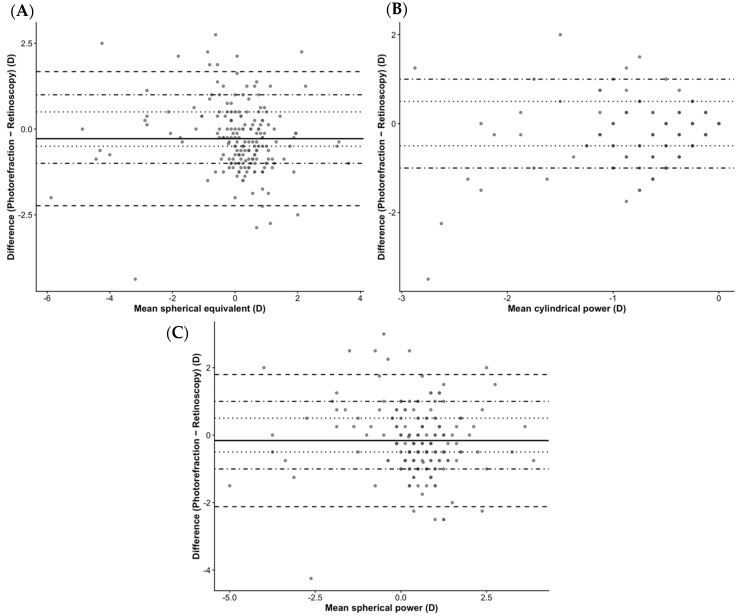
Agreement between photorefraction and retinoscopy for refractive parameters. Bland–Altman plots showing agreement between photorefraction and retinoscopy for (**A**) spherical power, (**B**) cylindrical power, and (**C**) spherical equivalent. Solid lines represent the mean inter-method difference, dashed lines indicate the 95% limits of agreement, dotted lines represent ±0.50 D, and dot-dashed lines represent ±1.00 D clinical thresholds.

Despite small mean inter-method differences, wide limits of agreement were observed for spherical power (−2.12 to +1.80 D), with a mean difference of −0.16 ± 1.00 D (*p* = 0.009) (Figure 2A).

Cylindrical power also showed wide limits of agreement (−1.48 to +1.00 D), with a mean difference of −0.24 ± 0.63 D (*p* < 0.001) (Figure 2B).

For spherical equivalent, a mild myopic shift was observed with photorefraction (mean difference −0.28 ± 1.00 D; *p* < 0.001), with limits of agreement ranging from −2.23 to +1.67 D (Figure 2C).

No significant proportional bias was observed for spherical equivalent (*p* = 0.126) or spherical power (*p* = 0.291). In contrast, a small but statistically significant proportional bias was detected for cylindrical power (*p* = 0.026).

Intraclass correlation coefficients indicated moderate-to-good agreement for spherical equivalent (ICC = 0.73; 95% CI: 0.64–0.79) and spherical power (ICC = 0.71; 95% CI: 0.63–0.77), whereas agreement for cylindrical power was poor (ICC = 0.46; 95% CI: 0.31–0.58).

Mean absolute error (MAE) was 0.80 D for spherical equivalent, 0.76 D for spherical power, and 0.48 D for cylindrical power. Only 45.1% of spherical equivalent measurements fell within ±0.50 D, indicating substantial variability at the individual level. Subgroup analyses revealed refractive-status-dependent variability, with emmetropic eyes showing the lowest MAE for spherical equivalent, followed by hyperopic and myopic eyes. Although mean differences were small, these findings indicate clinically relevant variability at the individual level. Importantly, despite statistically significant differences, the magnitude of agreement should be interpreted in a clinical context. The wide limits of agreement (up to approximately ±2.0 D) indicate that, for individual patients, measurements obtained by photorefraction may differ substantially from retinoscopy, potentially leading to clinically relevant discrepancies in refractive prescription.

### Subanalysis by Refractive Status

When stratified by refractive status based on retinoscopic spherical equivalent, inter-method agreement differed across refractive groups. Hyperopic eyes (*n* = 104) showed a larger negative mean inter-method difference (−0.67 ± 0.77 D) and wider limits of agreement (−2.19 to 0.85 D), indicating increased variability in this group. Emmetropic eyes (*n* = 49) exhibited the smallest mean difference (−0.11 ± 0.69 D) and the narrowest limits of agreement (−1.46 to 1.23 D), reflecting the most consistent agreement between methods. In contrast, myopic eyes (*n* = 40) showed a positive mean difference (0.53 ± 1.27 D), indicating a relative hyperopic shift in photorefraction compared to retinoscopy, with intermediate limits of agreement (−1.96 to 3.02 D).

Mean absolute error analyses further highlighted refractive-status-dependent variability, with emmetropic eyes showing the lowest MAE for spherical equivalent (0.54 D), followed by hyperopic (0.84 D) and myopic eyes (1.00 D). Concordance within ±0.50 D was highest in emmetropic eyes (63.3%), compared with hyperopic (37.5%) and myopic eyes (42.5%). Agreement within ±1.00 D followed a similar pattern, reaching 85.7% in emmetropic eyes and decreasing to 70.2% in hyperopic and 67.5% in myopic eyes. However, even in emmetropic eyes, agreement remained incomplete, indicating residual variability. Across the entire sample, agreement within ±0.50 D was observed in 45.1% of eyes for spherical equivalent, 51.3% for spherical power, and 74.0% for cylindrical power, indicating parameter-dependent variability in clinical concordance and reduced agreement for spherical components compared with cylindrical measurements. From a clinical perspective, the relatively low proportion of eyes within ±0.50 D, particularly in hyperopic and myopic groups, suggests that photorefraction may not provide sufficient accuracy for individual prescribing decisions, although it may still be appropriate for screening purposes.

## 4. Discussion

The present study assessed the agreement between non-cycloplegic retinoscopy and non-cycloplegic photorefraction in children aged 4–16 years and identified a small but statistically significant myopic shift in photorefraction measurements, together with wide limits of agreement and refractive-status-dependent variability. When interpreted in the context of existing literature, these findings align with a consistent body of evidence showing that automated and instrument-based refraction techniques (including photorefraction and autorefraction), while valuable, remain sensitive to methodological and physiological factors in pediatric populations.

Early comparative work by Choong et al. [28] provides an important reference framework for understanding these results. In their study, autorefractors showed a clear tendency toward minus overcorrection under non-cycloplegic conditions, leading to overdiagnosis of myopia, whereas agreement with subjective refraction improved markedly after cycloplegia. Although both techniques in our study were performed under non-cycloplegic conditions, the mild myopic bias observed with photorefraction is directionally consistent with the accommodative influences described by Choong et al. [28], suggesting that automated measurements may be more susceptible to residual accommodative activity than examiner-controlled retinoscopy, even when both are performed without cycloplegia. However, this apparent bias may be largely attributable to differences in accommodative demand between techniques, rather than reflecting a true device-related effect.

Building on this early evidence, later studies focusing on photoscreening devices have reported mixed findings regarding agreement and clinical interchangeability. Jesus et al. [29], for example, found statistically significant differences between Spot Vision Screening™ and subjective clinical refractometry under cycloplegia but considered these differences clinically negligible, supporting the use of photoscreeners as ancillary tools. In contrast, our results emphasize that, under non-cycloplegic conditions, modest mean differences may coexist with substantial individual-level variability. The wide limits of agreement and moderate intraclass correlation coefficients observed in our cohort suggest that photorefraction and retinoscopy should not be considered interchangeable for individual refractive assessment, particularly when measurements are obtained without pharmacological control of accommodation.

In addition, the combination of a relatively high mean absolute error (0.80 D) and a modest proportion of measurements within ±0.50 D (45.1%) suggests a broad distribution of inter-method differences, potentially deviating from normality, as reflected by the wide limits of agreement. This indicates substantial variability at the individual level. Such dispersion reinforces that agreement between methods cannot be adequately described by mean differences alone and highlights the importance of considering individual variability when interpreting non-cycloplegic measurements.

This apparent discrepancy across studies becomes more comprehensible when refractive status is taken into account. In line with Calvo-Maroto et al. [17], who demonstrated that non-cycloplegic measurements were reliable in myopic children but clinically inaccurate in hyperopic eyes due to incomplete accommodative relaxation, our data showed a clear refractive-status-dependent pattern. Hyperopic eyes exhibited the largest negative inter-method bias and the poorest agreement, whereas emmetropic eyes showed the smallest mean differences, lowest mean absolute error, and highest concordance rates. This pattern is also consistent with a non-cycloplegic classification bias, whereby residual accommodation may lead to underestimation of hyperopia and misclassification toward emmetropia or myopia. This consistency across studies reinforces the notion that hyperopia represents the most challenging refractive condition for objective measurements performed without cycloplegia, particularly when automated techniques are used.

Beyond refractive status, the role of device configuration has also emerged as a key determinant of agreement. Comparative studies involving handheld photorefraction devices and table-mounted autorefractors have repeatedly shown superior agreement between table-mounted devices and retinoscopy. Kurent et al. [30] reported that handheld autorefractors displayed greater variability and wider limits of agreement, particularly at higher refractive values. Our findings are concordant with this observation, as agreement deteriorated in eyes with higher refractive errors, despite relatively small mean differences. This suggests that refractive magnitude itself amplifies measurement variability, regardless of the specific automated technology employed.

Differences related to optical design have also been highlighted in comparisons between open-field and closed-field autorefractors (distinct from photorefraction devices). Kuo et al. [31] showed that open-field systems achieved closer agreement with retinoscopy, particularly for hyperopia detection in younger children, whereas closed-field instruments were more prone to accommodative artifacts. Although our study did not directly compare open- and closed-field devices, the refractive-status-dependent variability observed supports the broader conclusion that accommodative control and instrument design substantially influence measurement accuracy under non-cycloplegic conditions in pediatric refraction.

Finally, our findings regarding astigmatism further align with previous reports [31,32,33]. We observed lower agreement for cylindrical power, with significant proportional bias and reduced ICC values. This mirrors multiple studies showing that photoscreeners (photorefraction devices) and handheld autorefractors are less reliable for estimating cylindrical components and astigmatic axis than for spherical measurements. Even in the absence of cycloplegia, cylindrical measurements appear particularly susceptible to inter-method variability, underscoring the need for cautious interpretation when astigmatism plays a critical role in clinical decision-making.

Several limitations of the present study should be acknowledged. First, although the overall sample size was adequate to detect statistically significant inter-method differences, no formal sample size or power calculation was performed a priori. Subgroup analyses by refractive status resulted in smaller sample sizes, particularly in the myopic group, which may have limited statistical power and contributed to wider confidence intervals and limits of agreement in some comparisons. Therefore, subgroup findings should be interpreted with caution, particularly in the myopic group. Second, the absence of cycloplegic measurements represents an important limitation. In pediatric clinical practice, cycloplegia fundamentally influences the interpretation of refractive findings, particularly in hyperopic children, by minimizing accommodative responses and revealing latent hyperopia. Therefore, the agreement observed in this study reflects non-cycloplegic conditions that are more representative of screening environments than of definitive refractive assessment. Importantly, the two techniques differed systematically in accommodative demand due to fixation distance (approximately 4 m for retinoscopy vs. 1 m for photorefraction), which may have contributed to the observed myopic shift. Thus, part of the inter-method difference may reflect differential accommodative responses rather than true disagreement between techniques. Third, examiners were not masked to the results of the alternative method, which may have introduced measurement bias and potentially led to an overestimation of agreement between methods. In addition, the order of testing was not randomized and followed routine clinical practice, which may have introduced an order effect. In clinical settings, prior knowledge of refractive findings can unintentionally influence subsequent measurements, potentially reducing inter-method variability and affecting agreement estimates. Fourth, only a single measurement per method was recorded for each participant. In pediatric populations, where cooperation and fixation stability may vary, repeatability is a key aspect of measurement reliability. The absence of repeated measurements limits the ability to assess intra-method variability and the stability of the measurements obtained. Furthermore, only one valid measurement per method was retained, and the number of attempts required to obtain a valid photorefraction measurement, as well as the proportion of excluded measurements, was not systematically recorded, preventing evaluation of potential exclusion bias. Fifth, the study was restricted to a pediatric population aged 4–16 years; therefore, the findings may not be generalizable to younger preschool children or adolescents, in whom accommodative behavior and testability may differ. Sixth, only a single photorefraction device was evaluated, and results may not be directly extrapolated to other photoscreeners or autorefractors with different optical designs or measurement algorithms. Seventh, astigmatism was analyzed using scalar cylindrical power rather than vector components (J0 and J45). Although vector analysis is mathematically preferable when reliable axis measurements are available, the astigmatic axis in the present study was determined by non-cycloplegic clinical retinoscopy using trial lenses. In pediatric populations, small uncertainties in axis estimation may substantially affect vector components; therefore, we considered that vector decomposition could introduce additional noise rather than improve analytical robustness. Finally, subjective refraction was not included as a comparative method, which may have provided complementary clinical insight into functional refractive outcomes.

Future research should address these limitations by including larger, age-stratified samples and by comparing multiple automated devices under standardized cycloplegic and non-cycloplegic conditions. Longitudinal studies would be particularly valuable to determine whether small but systematic inter-method differences translate into clinically meaningful outcomes over time, especially in hyperopic children. Further work should also explore the impact of automated refraction-based prescriptions on accommodative demand and visual development. Finally, integrating automated measurements with clinical decision algorithms may help optimize the role of photorefraction as a screening tool while preserving cycloplegic retinoscopy as the reference standard for definitive pediatric refractive care.

## 5. Conclusions

This study demonstrates that photorefraction shows a small but statistically significant myopic shift when compared with non-cycloplegic retinoscopy in children aged 4–16 years, which is likely primarily explained by systematic differences in accommodative demand between techniques. Although mean inter-method differences were modest, the wide limits of agreement observed indicate clinically relevant variability at the individual level. Agreement between methods was highest for spherical equivalent and spherical power and lowest for cylindrical power, highlighting parameter-dependent performance. Importantly, inter-method agreement varied according to refractive status, with emmetropic eyes showing the most consistent results and hyperopic eyes exhibiting the greatest variability and tendency toward underestimation.

These findings support the use of photorefraction as a practical and efficient tool for pediatric vision screening and population-based assessments under non-cycloplegic conditions. However, its role should be clearly distinguished from that of diagnostic or prescribing tools. Photorefraction should not be used as a standalone method for individualized refractive prescriptions, particularly in hyperopic children or when accurate assessment of astigmatism is required. Instead, it should be considered a screening instrument to identify children who require further evaluation. Cycloplegic retinoscopy remains essential for definitive refractive assessment and clinical decision-making in pediatric practice.

## Figures and Tables

**Table 1:** Descriptive statistics of refractive components obtained by retinoscopy and photorefraction.

Parameter	Measurement	Retinoscopy	Photorefraction
**Spherical equivalent (D)**	Mean ± SD	0.23 ± 1.43	−0.05 ± 1.33
	Median [IQR]	0.50 [1.25]	0.00 [1.00]
	Range (D)	−5.50 to 4.12	−6.88 to 3.25
**Sphere (D)**	Mean ± SD	0.47 ± 1.35	0.31 ± 1.28
	Median [IQR]	0.50 [1.25]	0.25 [1.25]
	Range (D)	−5.00 to 4.25	−5.75 to 3.75
**Cylinder (D)**	Mean ± SD	−0.48 ± 0.58	−0.72 ± 0.67
	Median [IQR]	−0.25 [0.75]	−0.50 [0.75]
	Range (D)	−3.50 to 0.00	−4.50 to 0.00

IQR: interquartile range.; D: Diopters; SD: Standard deviation.

## Data Availability

The original contributions presented in this study are included in the article. Further inquiries can be directed to the corresponding author.

## Supplementary Materials

The following supporting information can be downloaded at https://www.mdpi.com/article/10.3390/life16040678/s1. Supplementary Material S1. Individual participant data including demographic and refractive variables measured by retinoscopy and photorefraction.
