# Supplementary material for: Agreement Between Non-Cycloplegic Photorefraction and Retinoscopy in Pediatric Refraction
Source: Life (Basel). 2026 Apr 16;16(4):678. doi: 10.3390/life16040678 (PMC13117380; doi:10.3390/life16040678)
Supplement: Supplementary file 1 [file life-16-00678-s001.zip › life-4247598-supplementary.pdf]

Supplementary Material S1. Individual participant data including demographic and refractive variables measured by retinoscopy and photorefraction

ID	Age	Sex	Sphere (Retinoscopy)	Cylinder (Retinoscopy)	Spherical equivalent (Retinoscopy)	Sphere (Photorefraction)	Cylinder (Photorefraction)	Spherical equivalent (Photorefraction)
1	6	M	0.75	0	0.75	1.25	-1	0.75
2	6	F	1.5	0	1.5	0.25	0	0.25
3	6	M	0.5	0	0.5	0	-0.5	-0.25
4	5	F	3.5	-0.5	3.25	1.25	-1	0.75
5	6	F	-0.25	0	-0.25	0	0	0
6	6	F	0	-0.25	-0.125	0	-0.25	-0.125
7	6	M	-0.25	-0.5	-0.5	0.75	-0.5	0.5
8	6	M	0.5	0	0.5	0.5	0	0.5
9	7	M	0	-0.5	-0.25	1	-0.5	0.75
10	7	F	0.25	-0.5	0	0.25	-0.75	-0.125
11	7	F	1	0	1	0.25	-0.75	-0.125
12	7	M	2.5	0	2.5	0	-0.5	-0.25
13	7	F	1	0	1	-0.25	-0.25	-0.375
14	7	F	1	-1.5	0.25	-0.25	-0.75	-0.625
15	6	F	0	0	0	-0.25	0	-0.25
16	7	M	0.5	0	0.5	-0.5	-0.25	-0.625
17	5	M	1	-1	0.5	1	-0.5	0.75
18	5	M	1.5	0	1.5	0.75	-0.5	0.5
19	5	F	0.5	0	0.5	0.5	0	0.5
20	5	F	1.5	0	1.5	0.75	-1	0.25
21	5	M	1.5	0	1.5	1.5	-0.75	1.125
22	9	M	0.5	0	0.5	0	-0.5	-0.25
23	9	F	1.25	-0.25	1.125	0.5	-0.5	0.25
24	9	F	1	0	1	0.75	-1.25	0.125
25	9	F	1	0	1	-0.5	0	-0.5
26	7	M	0.75	0	0.75	0.75	-0.75	0.375
27	7	M	0.75	-0.5	0.5	0	-1	-0.5

28	7	F	0	-0.25	-0.125	-0.25	-0.25	-0.375
29	7	F	1.75	0	1.75	1	-0.75	0.625
30	9	F	-0.5	0	-0.5	-0.5	-0.25	-0.625
31	9	M	0	0	0	1	-1.25	0.375
32	10	F	0.25	-0.25	0.125	0	-0.25	-0.125
33	9	M	0.75	-0.25	0.625	-0.25	0	-0.25
34	7	F	2.25	-0.25	2.125	-0.25	-1	-0.75
35	7	M	1.5	-0.5	1.25	0	0	0
36	8	F	0.5	-0.25	0.375	-0.25	-0.25	-0.375
37	8	F	0.5	-0.5	0.25	-0.25	0	-0.25
38	8	M	-1.5	-0.25	-1.625	-1	0	-1
39	8	F	1	0	1	0	-0.5	-0.25
40	8	F	0.5	0	0.5	0	0	0
41	8	F	1	0	1	0.75	-1.25	0.125
42	8	M	-1	-0.5	-1.25	-0.25	-1.25	-0.875
43	5	F	2.5	-1	2	0.5	-0.75	0.125
44	4	M	1	0	1	0.75	-0.5	0.5
45	5	M	0.75	-0.25	0.625	1.25	0	1.25
46	5	M	2	-1.75	1.125	1.25	-1.25	0.625
47	5	F	-0.25	0	-0.25	0	0	0
48	5	M	-0.25	0	-0.25	0.25	0	0.25
49	5	M	1.25	-0.75	0.875	0.75	-0.5	0.5
50	7	M	1.25	-0.25	1.125	0.25	-0.25	0.125
51	7	M	1	-0.25	0.875	0	-0.5	-0.25
52	7	M	1.75	-1.5	1	0.25	0	0.25
53	7	F	1.75	0	1.75	0.25	-0.5	0
54	8	M	0.75	-1	0.25	0.5	-0.5	0.25
55	8	F	1	-0.25	0.875	0.5	-0.25	0.375
56	8	M	-0.25	-1	-0.75	0.5	-1.5	-0.25
57	9	F	0.25	-1	-0.25	1.5	-1	1
58	8	M	0.25	-1	-0.25	0.75	-1.5	0
59	8	M	0	-1	-0.5	-0.25	-0.5	-0.5

60	11	M	0.75	0	0.75	0.25	-1.5	-0.5
61	9	F	-0.25	-1	-0.75	1.5	-1.25	0.875
62	4	M	3	-1	2.5	2	-1.5	1.25
63	5	M	1.25	-1	0.75	-0.25	-0.75	-0.625
64	4	M	0.25	-0.5	0	1.5	-0.5	1.25
65	5	M	0.75	0	0.75	0.25	-0.25	0.125
66	4	F	1.25	-1	0.75	1.25	-0.75	0.875
67	9	M	0.75	0	0.75	0	-0.5	-0.25
68	9	M	1.05	-0.5	0.8	0.25	-0.75	-0.125
69	12	F	1	-0.75	0.625	0.5	-1.25	-0.125
70	9	F	1	0	1	0.5	-1.25	-0.125
71	8	F	0.5	-0.25	0.375	0	-0.5	-0.25
72	6	M	-0.5	0	-0.5	0.25	-0.5	0
73	6	F	0.5	0	0.5	0.25	-0.5	0
74	9	M	1.5	0	1.5	1	-1	0.5
75	6	M	-1.5	-0.5	-1.75	0.25	-0.25	0.125
76	6	M	1.5	-1.5	0.75	1	-0.5	0.75
77	6	F	2	-1.5	1.25	1.5	-3	0
78	6	M	0.5	-3.5	-1.25	0.75	-2.25	-0.375
79	6	F	0.5	0	0.5	0.75	-0.25	0.625
80	6	M	1.5	-1	1	1.75	-1.25	1.125
81	6	F	1.5	-0.5	1.25	2	-0.75	1.625
82	6	F	0.5	0	0.5	0.25	-0.5	0
83	5	F	1.5	-2	0.5	2	-1.75	1.125
84	4	M	2.5	-1	2	2	-1	1.5
85	5	F	2	0	2	2.75	-1.75	1.875
86	5	M	1.5	-1	1	3.5	-0.5	3.25
87	5	F	2.5	-1	2	0	-0.5	-0.25
88	5	F	-0.5	-1	-1	0.5	-0.5	0.25
89	6	M	0.5	-1	0	1	-4.5	-1.25
90	5	M	0.5	0	0.5	2	-0.5	1.75
91	5	F	2	0	2	2.25	-1	1.75

92	5	M	1.5	0	1.5	0.25	0	0.25
93	5	M	1.5	0	1.5	1	-0.75	0.625
94	7	M	0.5	0	0.5	1	0	1
95	8	F	0.75	0	0.75	1.75	-1	1.25
96	7	F	0.75	-0.75	0.375	-0.25	-0.5	-0.5
97	8	M	-0.5	-0.5	-0.75	0	-0.75	-0.375
98	8	F	-0.5	-0.25	-0.625	0.5	-0.75	0.125
99	9	M	0.5	-0.5	0.25	0.75	-1.5	0
100	9	F	0.5	0	0.5	0.25	-0.5	0
101	9	F	1.5	0	1.5	-0.25	-0.25	-0.375
102	8	F	0.5	-1	0	1.75	-0.75	1.375
103	9	M	-0.5	-1	-1	-4.75	-1.25	-5.375
104	7	M	0.5	-0.25	0.375	-0.5	-0.25	-0.625
105	7	M	0.75	-0.25	0.625	0.25	0	0.25
106	7	F	1.25	-0.5	1	0.25	-0.5	0
107	10	M	-0.25	-0.25	-0.375	0.5	-0.75	0.125
108	6	F	0.5	-0.5	0.25	1.75	-1	1.25
109	10	F	-2.5	-1	-3	-1.5	-2.25	-2.625
110	14	M	-2	-1.75	-2.875	-1.25	-3	-2.75
111	15	M	0.5	-1	0	0.25	-1.25	-0.375
112	10	F	4.25	-0.25	4.125	3.5	-0.75	3.125
113	14	M	1.5	-0.5	1.25	1.25	0	1.25
114	14	M	-3.5	-1	-4	-4	-1.25	-4.625
115	14	F	0.5	-0.25	0.375	-0.25	-0.75	-0.625
116	14	M	-1	-0.5	-1.25	-0.75	-0.25	-0.875
117	15	M	0.5	-0.5	0.25	-0.25	-1.5	-1
118	12	M	1.5	0	1.5	0.5	-0.25	0.375
119	12	M	-5	-1	-5.5	-3	0	-3
120	14	M	-0.5	0	-0.5	0.5	-0.5	0.25
121	14	M	0	-0.25	-0.125	0.75	-0.75	0.375
122	12	M	1	-0.5	0.75	1	0	1
123	13	F	0	0	0	-0.75	-0.25	-0.875

124	14	F	-2.75	-0.25	-2.875	-0.25	-1	-0.75
125	15	M	0	-2.5	-1.25	0	-0.5	-0.25
126	16	F	-1.5	-0.25	-1.625	-1.25	-1.25	-1.875
127	9	M	1.5	-1.25	0.875	0.25	-0.5	0
128	12	M	1.75	0	1.75	1.5	-1.5	0.75
129	14	F	0	-0.5	-0.25	-0.25	-0.5	-0.5
130	11	F	-3	0	-3	-2.5	-0.5	-2.75
131	11	M	-4.25	-1.25	-4.875	-5.75	-2.25	-6.875
132	12	F	0.5	-0.25	0.375	-0.5	-0.5	-0.75
133	14	F	-2.5	-2.25	-3.625	-3.75	-1.25	-4.375
134	13	M	0.25	-0.5	0	0.2	-1.25	-0.425
135	9	M	1.75	-0.75	1.375	0.75	0	0.75
136	11	M	0	-0.25	-0.125	-1.5	-0.25	-1.625
137	11	F	1.5	-1.5	0.75	2	-3.75	0.125
138	13	M	0.5	-0.5	0.25	0.75	-0.75	0.375
139	13	M	1.75	-0.25	1.625	1	-0.5	0.75
140	14	F	0.5	-0.5	0.25	0.5	-0.5	0.25
141	13	M	1	0	1	0.75	0	0.75
142	11	F	-1	-1	-1.5	-1.5	-0.75	-1.875
143	11	M	-2.25	-0.25	-2.375	-1.5	-0.75	-1.875
144	14	M	1	-0.5	0.75	0	-0.75	-0.375
145	14	F	1	0	1	-0.5	0	-0.5
146	11	F	0	-0.5	-0.25	-0.25	0	-0.25
147	9	M	-2.5	-1.75	-3.375	-1.25	-2	-2.25
148	14	F	0.5	-0.5	0.25	0	-0.25	-0.125
149	10	F	1.5	-1	1	-0.75	-0.5	-1
150	12	F	0	-0.5	-0.25	0.25	-0.5	0
151	10	F	0.25	0	0.25	-0.25	0	-0.25
152	10	M	0.5	-1	0	1.25	-1.75	0.375
153	10	M	0.75	-0.25	0.625	0	-0.75	-0.375
154	10	M	0	0	0	-0.75	0	-0.75
155	11	F	0	-0.75	-0.375	0	-1.25	-0.625

156	11	M	0	-0.5	-0.25	-0.25	-0.5	-0.5
157	10	F	0.5	0	0.5	-0.25	0	-0.25
158	11	M	0.5	-1	0	1.5	-1.25	0.875
159	11	M	1	-0.5	0.75	-0.5	0	-0.5
160	13	F	2	-0.75	1.625	3.5	-1.25	2.875
161	12	F	-1	-0.5	-1.25	-1	-0.5	-1.25
162	8	F	-1	0	-1	1.5	-0.75	1.125
163	10	M	3.5	0	3.5	3.75	-1.25	3.125
164	7	M	-3	-2	-4	-3.75	-2.25	-4.875
165	5	F	2	0	2	2	-0.25	1.875
166	5	M	-0.5	0	-0.5	0	-0.25	-0.125
167	8	F	1.25	-0.5	1	0.5	-0.25	0.375
168	8	M	1	-0.25	0.875	1.25	-1	0.75
169	5	M	-2	0	-2	-1.75	-0.75	-2.125
170	8	M	3.5	0	3.5	3	0	3
171	9	M	1	0	1	1.25	-1	0.75
172	8	M	2	0	2	1.5	-0.75	1.125
173	7	M	0.5	0	0.5	0	-0.25	-0.125
174	7	M	-1	0	-1	0	0	0
175	7	F	-2	0	-2	0.5	-0.5	0.25
176	10	F	0.5	0	0.5	0.25	-0.5	0
177	6	F	0.75	-0.25	0.625	0.25	-0.5	0
178	6	F	1.25	-0.5	1	1.25	-0.75	0.875
179	6	M	-2	0	-2	1	-0.5	0.75
180	6	F	1	-0.5	0.75	1.25	-0.5	1
181	7	M	-0.5	-1.5	-1.25	0.25	-0.25	0.125
182	6	M	1.5	-1.5	0.75	0.5	-0.75	0.125
183	6	F	1	0	1	0.5	-0.5	0.25
184	6	F	-1.5	0	-1.5	0.75	-0.75	0.375
185	5	M	1	0	1	0.75	-0.75	0.375
186	5	M	1.25	0	1.25	0.75	0	0.75
187	6	F	0.25	-0.25	0.125	0.75	-0.25	0.625

188	5	M	0.75	-1.25	0.125	0.5	-1	0
189	8	F	1.25	-1	0.75	1.25	-1.25	0.625
190	7	M	-3.75	-2.25	-4.875	-3.75	-2.25	-4.875
191	7	F	0.25	-0.25	0.125	0.25	-0.25	0.125
192	10	F	0.5	-1	0	0.5	-1	0
193	5	F	0.5	0	0.5	0.25	-0.25	0.125
